# Low-dose carbon-based nanoparticle-induced effects in A549 lung cells determined by biospectroscopy are associated with increases in genomic methylation

**DOI:** 10.1038/srep20207

**Published:** 2016-02-02

**Authors:** Junyi Li, Meiping Tian, Li Cui, John Dwyer, Nigel J. Fullwood, Heqing Shen, Francis L. Martin

**Affiliations:** 1 Centre for Biophotonics, LEC, Lancaster University, Lancaster LA1 4YQ, UK; 2 Key Lab of Urban Environment and Health, Institute of Urban Environment, Chinese Academy of Sciences, Xiamen 361021, China; 3 Division of Biomedical and Life Sciences, Faculty of Health and Medicine, Lancaster University, Lancaster LA1 4YQ, UK

## Abstract

Nanotechnology has introduced many manufactured carbon-based nanoparticles (CNPs) into our environment, generating a debate into their risks and benefits. Numerous nanotoxicology investigations have been carried, and nanoparticle-induced toxic effects have been reported. However, there remain gaps in our knowledge, primarily regarding mechanism. Herein, we assessed the global alterations induced by CNPs in A549 lung cells using biospectroscopy techniques, including attenuated total reflection Fourier-transform infrared (ATR-FTIR) spectroscopy and surface-enhanced Raman spectroscopy (SERS). A549 cells were treated with fullerene (C_60_), long or short multi-walled carbon nanotubes, or single-walled carbon nanotubes at concentrations of 0.1 mg/L, 0.01 mg/L and 0.001 mg/L. Exposed cells were then analysed by ATR-FTIR spectroscopy and SERS. Spectra were pre-processed *via* computational analysis, and information on biochemical alterations in exposed cells were identified. Additionally, global DNA methylation levels in cells exposed to CNPs at 0.1 mg/L were determined using HPLC-MS and genetic regulators (for DNA methylation) were checked by quantitative real-time RT-PCR. It was found that CNPs exert marked effects in A549 cells and also contribute to increases in global DNA methylation. For the first time, this study highlights that real-world levels of nanoparticles can alter the methylome of exposed cells; this could have enormous implications for their regulatory assessment.

With rapid developments in nanotechnology, numerous nanomaterials are entering into the environment. Consequently, potential exposures to nanomaterials are likely to have increased dramatically in recent times. Particularly, carbon-based materials (CBMs) are currently widely used in both daily life applications and industry^1^,^2^,^3^,^4^. Thus, there is an urgent need to understand potential health hazards related to their exposure prior to increasing usage^5^,^6^.

CBMs are one of the most attractive nanomaterials due to their unique physicochemical properties depending on different forms, such as fullerenes (C_60_), single- or multi-walled carbon nanotubes (SWCNTs or MWCNTs) [see Electronic Supporting Information (ESI) Figure S1]. Numerous investigations have been carried out to assess these carbon nanoparticles (CNPs), and toxic effects both *in vitro* and *in vivo* have been reported^7^,^8^,^9^,^10^. Commonly, generation of reactive oxygen species (ROS) is considered a major factor involved in the toxicity of CNPs^11^. However, there remain knowledge gaps between nanotoxicology and real-world effects of CNPs^12^. Quite a few conventional toxicology assays appear limited in their applicability towards assessment of nanotoxicity (*e.g.*, nanoparticles may interact with the dye in the MTT or LDH assays; nanoparticles might well interfere with enzymatic immunoassays); thus novel testing methodologies are required^13^. Such novel testing methodologies could verify existing toxicology assays whilst leveraging additional insights.

Raman or attenuated total reflection Fourier-transform infrared (ATR-FTIR) spectroscopy techniques are vibrational spectroscopic approaches capable of deriving biochemical information from biological samples^14^. Vibrational spectroscopy approaches, in a reagent-free fashion, have the capability to inexpensively analyse biological samples; these methods have been used to investigate cancer and assess toxic effects of environmental pollution^15^,^16^,^17^,^18^,^19^,^20^,^21^. Infrared (IR) spectroscopy is the measurement of the energy absorption of the chemical bond vibrational or rotational movement occurring at a specific energy level. This generates spectra with peaks representing chemical bonds as well as the vibration/rotations of groups of atoms in a sample. The mid-IR region (1800–900 cm^−1^), known as the “biochemical fingerprint” region, is where the majority IR absorption occurs for biochemical structures. As a scattering technique, Raman spectroscopy provides a complementary spectrochemical approach. Surface-enhanced Raman spectroscopy (SERS) is a variation of the technique that exhibits high detection sensitivity in generating fingerprint spectra^22^,^23^. Its detection sensitivity relies on strong electromagnetic enhancement, typically produced by Ag or Au nanoparticles (NPs); Raman signals of molecules close to these NPs can be enhanced by many orders of magnitude^24^,^25^. This makes SERS an ultrasensitive tool, down to the single molecule level^26^; however, there are limitations associate with this approach relating to a lack of reproducibility and/or consistency in the level of enhancement.

Our objective herein was to perform an assessment of four CNPs (C_60_, long or short MWCNTs, single-walled CNTs) to induce cellular alterations using biospectroscopic techniques (Fig. 1). Due to their size, such CNPs can readily become airborne, inhaled and induce pulmonary injury. Hence, the human epithelial-like lung A549 cell line was employed. For *in vitro* or *in vivo* toxicological tests, dose is a key parameter to understanding real-world effects. Although acute effects are more likely to be observed following high-dose exposures, recording the consequences of low-dose levels over a lifetime is necessary to understand possible risks of CNPs. Thus, cells were exposed to CNPs at ppb levels at a dose range of 0.1 mg/L, 0.01 mg/L and 0.001 mg/L. Following exposure, ATR-FTIR spectroscopy and SERS were employed. Also, global DNA methylation levels in cells exposed to 0.1 mg/L CNPs were determined by HPLC-MS; disruption of methylation in cells is linked to diseases such as cancer^27^,^28^,^29^. This study aims to assess the ability of CNPs to induce effects at ppb levels.

## Results

### ATR-FTIR spectral dataset

**Figure 2:**
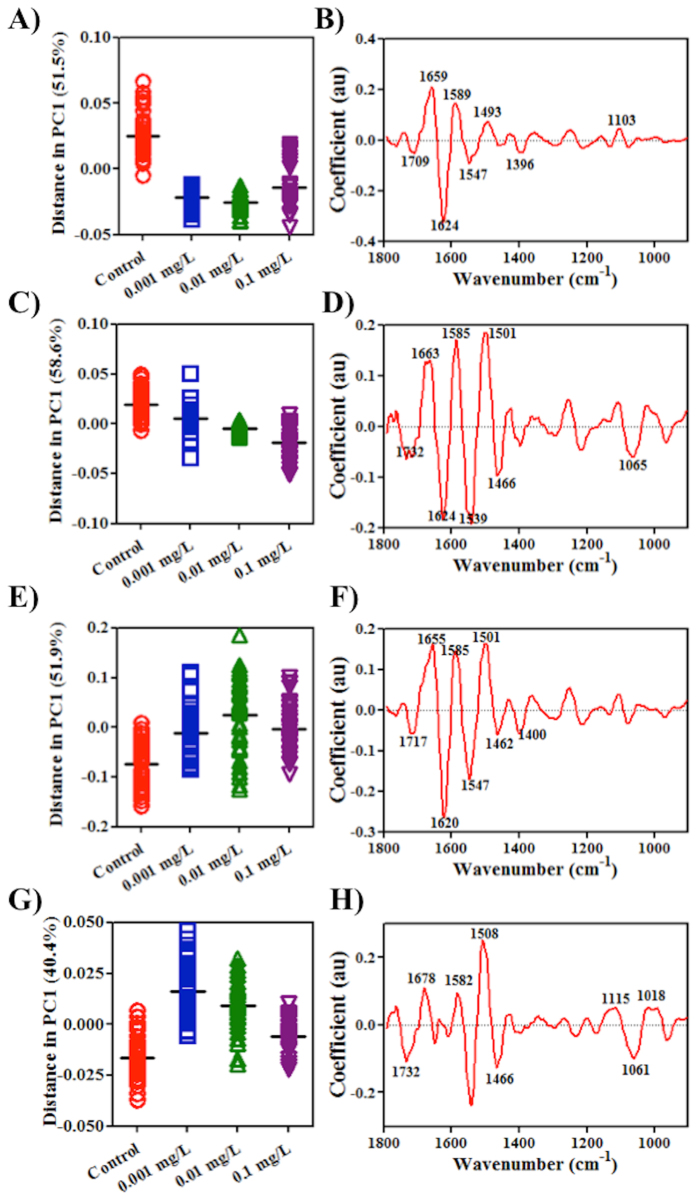
One-dimensional (1-D) PCA scores plots in PC1 space of attenuated total reflection Fourier-transform infrared (ATR-FTIR) spectral dataset derived from A549 cells exposed to carbon nanoparticles at each concentration *vs.* vehicle control. Their corresponding loadings plots are on the right. Panels represent: (**A, B**) C_60_ fullerene; (**C, D**) long MWCNTs; (**E, F**) short MWCNTs; and, (**G, H**) SWCNTs.

The spectra derived following ATR-FTIR spectroscopy were inputted into principal component analysis (PCA) following pre-processing. PCA scores plots allow one to visualise clustering among classes (see ESI Figure S2). This allows observation of a dose-response in one-dimensional (1-D) scores plots in PC1 space (Fig. 2); scores in PC1 space contribute most variance in the spectral data. To determine whether the treatment groups were significantly different from the corresponding control group, repeated-measures one-way analysis of variance (ANOVA) with Dunnett’s *post-hoc* tests were conducted to examine the treated *vs*. control cells in PC1 space (see ESI Table S4). It shows that in all treatment groups, CNP-induced effects observed differ significantly (*P* < 0.05) from the control group.

Loadings plots derived from the PC1 space identify the distinguishing wavenumbers corresponding to the most important variances for each CNP treatment. The eight wavenumbers contributing most in each loadings plot are marked. For each CNP exposure, the distinguishing wavenumbers ranked as follows: 1624, 1658, 1589, 1547,1493, 1709, 1396 and 1103 cm^−1^ in C_60_ treatment group; 1539, 1501, 1624, 1585, 1662, 1466, 1732 and 1065 cm^−1^ in long MWCNTs group; 1620, 1547, 1501, 1655, 1585, 1462, 1717 and 1400 cm^−1^ in short MWCNTs group; and, 1508, 1466, 1678, 1732, 1061, 1582, 1018 and 1115 cm^−1^ in SWCNTs group, respectively. These wavenumbers contribute most to segregation in PC1 space (for tentative assignments, see ESI Table S1).

**Figure 3:**
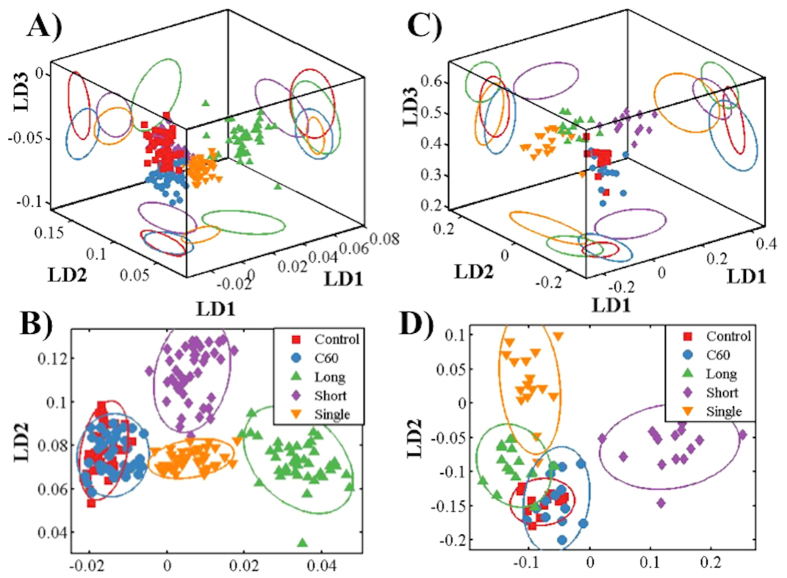
Scores plots by principal component analysis-linear discriminant analysis (PCA-LDA) derived from spectral dataset of the A549 cells exposed to CNPs at 0.1 mg/L *vs.* vehicle control (*90% confidence ellipsoids*). (**A**) Three-dimensional (3-D); and (**B**) 2-D PCA-LDA scores plots from the attenuated total reflection Fourier-transform infrared (ATR-FTIR) spectral dataset. (**C**) 3-D; and (**D**) 2-D PCA-LDA scores plots from the SERS spectral dataset. C_60_, C_60_ fullerene; Long, long MWCNTs; Short, short MWCNTs; and, Single, SWCNTs.

**Figure 4:**
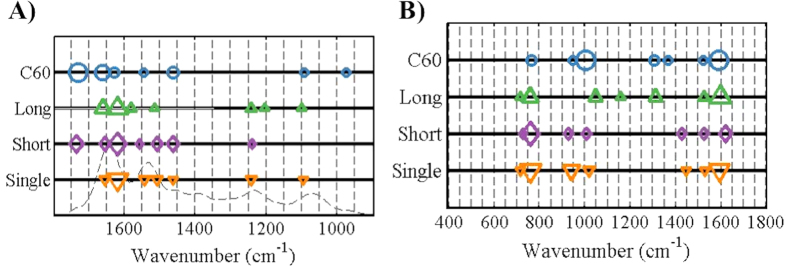
Cluster vectors peak plots following principal component analysis-linear discriminant analysis (PCA-LDA) indicating the wavenumber basis for segregation corresponding to A549 cells exposed to CNPs at 0.1 mg/L. Each cell population exposed to a different CNP was compared with the vehicle control. The size of the symbol in cluster vectors peak plot is proportional to the height of the corresponding peaks in the cluster vectors plots, which are relative to the extent of biochemical alteration *vs.* vehicle control. (**A**) Cluster vectors peak plot derived from attenuated total reflection Fourier-transform infrared (ATR-FTIR) spectral dataset; and (**B**) Cluster vectors peak plot derived from SERS spectral dataset. C_60_, C_60_ fullerene; Long, long MWCNTs; Short, short MWCNTs; and, Single, SWCNTs.

We then set out to compare cellular responses when cells were exposed to different CNPs at 0.1 mg/L, *i.e.*, the highest exposure examined in this study. Following PCA, linear discriminant analysis (LDA) was applied; this supervised analysis takes category information into account. Scores in the first three LD spaces were used for visualisation (Fig. 3A,B). Separation between groups was significant. Moreover, cluster vectors plots derived from PCA-LDA highlighted biochemical alterations in cells induced by the different CNPs (see ESI Figure S4). To simplify the identification of the most pronounced wavenumbers responsible for alterations, a peak detector was employed and a cluster vectors peak plot was generated (Fig. 4A; tentative assignments for the first seven wavenumbers are listed in ESI Table S3).

### SERS spectral dataset

**Figure 5:**
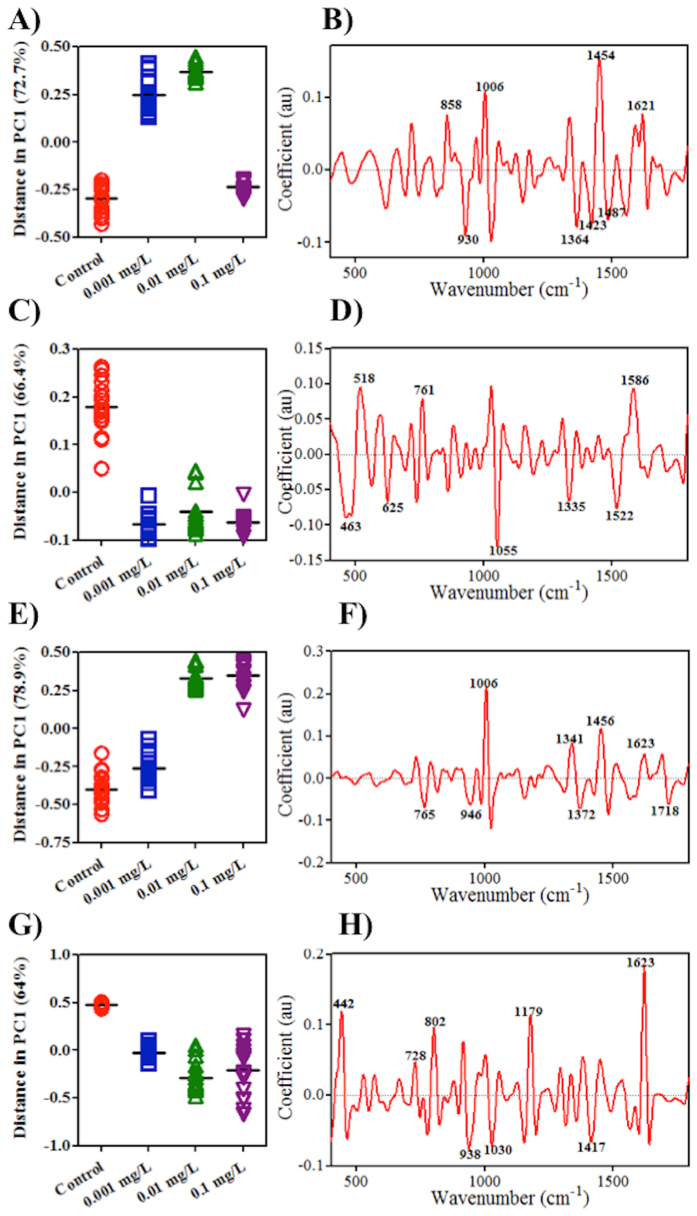
One-dimensional (1-D) principal component analysis (PCA) scores plots in PC1 space of SERS spectral dataset derived from A549 cells exposed to carbon nanoparticles at each concentration *vs.* vehicle control. Their corresponding loadings plots are on the right. Panels represent: (**A, B**) C_60_ fullerene; (**C, D**) long MWCNTs; (**E, F**) short MWCNTs; and, (**G, H**) SWCNTs.

Following a similar workflow, SERS spectral data was pre-processed prior to further analysis. The PC1 scores plots show significant separation between treatment groups *vs.* control (Fig. 5; statistical results in ESI Table S4). Similarly, the top eight distinguishing wavenumbers in the loadings plots were: 1454, 1006, 930, 1364, 1621, 1423, 858 and 1487 cm^−1^ for C_60_ exposure; 1055, 518, 1586, 463, 761, 1522, 625 and 1335 cm^−1^ for long MWCNTs exposure; 1006, 1456, 1341, 1372, 765, 946, 1718 and 1623 cm^−1^ for short MWCNTs exposure; and, 1623, 442, 1179, 802, 938, 1030, 1417 and 728 cm^−1^ for SWCNTs exposure (tentative assignments are listed in ESI Table S2).

PCA-LDA can give rise to visualisation in either 2-D or 3-D scores plots (Fig. 3C,D). Significant segregation was observed between CNPs treatment groups *vs.* control. Furthermore, a cluster vectors peak plot displayed the main biochemical alterations induced by different CNPs (Fig. 4B; tentative assignments of the first seven wavenumbers are listed in ESI Table S3).

### Global DNA methylation levels determined by HPLC-MS

**Figure 6:**
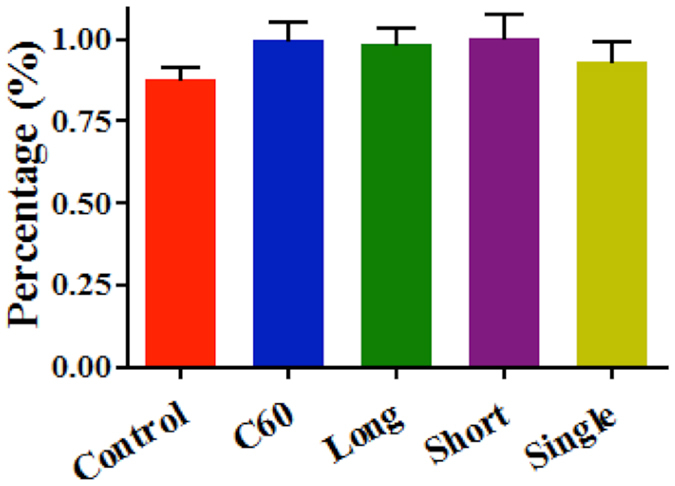
Global DNA methylation levels in A549 cells exposed to CNPs at 0.1 mg/L or without treatment, *i.e.*, control. Values are expressed as mean ± SD (*n* = 3). C_60_, C_60_ fullerene; Long, long MWCNTs; Short, short MWCNTs; and, Single, SWCNTs.

Global methylation levels in A549 cells were observed to increase following CNPs (0.1 mg/L) exposure. Mean levels of global genomic methylation in the control group was 0.88%, while mean values increased to 0.9967%, 0.9867%, 1.003% and 0.93% following exposure to C_60_, long or short MWCNTs, and SWCNTs, respectively (Fig. 6). Global DNA methylation level was significantly elevated by C_60_ (*P* < 0.05) or short MWCNTs (*P* < 0.05) treatment (see ESI Table S5).

### Quantitative real-time RT-PCR

**Figure 7:**
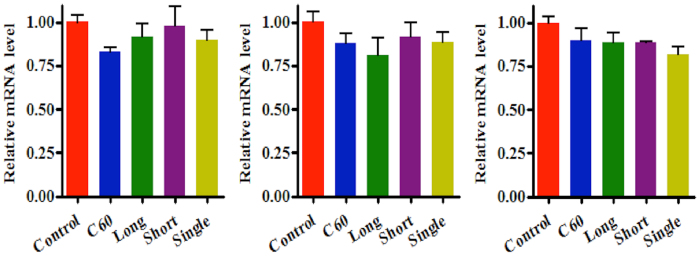
Quantitative real-time RT-PCR analysis of gene expression in A549 cells exposed to CNPs at 0.1 mg/L - candidate genes were: (A) *DNMT1* (B) *DNMT3a*; and, (C) *DNMT3b*. Total RNA was isolated, reverse transcribed, and amplified with the specific primers. Relative quantification of each gene expression level was normalized according to *GAPDH* expression. The data of exposure groups were calibrated to the control values (control = 1); values are expressed as mean ± SD (*n* = 3). C_60_, C_60_ fullerene; Long, long MWCNTs; Short, short MWCNTs; and, Single, SWCNTs.

Quantitative analysis was used to assess gene expression levels of DNMTs in A549 cells exposed to CNPs (0.1 mg/L). Post-exposure, *DNMT1* expression levels were determined as 1.001- (C_60_), 0.8905- (long MWCNTs), 0.9297- (short MWCNTs), and 0.9284-fold (single-walled CNTs) of the control group. Similarly, *DNMT3a* expression was determined as 1.036- (C_60_), 0.7928- (long MWCNTs), 0.9018- (short MWCNTs), and 0.9606-fold (single-walled CNTs) of the control group, whilst *DNMT3b* levels decreased to 0.9849- (C_60_), 0.8198- (long MWCNTs), 0.8171- (short MWCNTs), and 0.7553-fold (single-walled CNTs) of the control group (Fig. 7). Generally, a down-regulating tendency in *DNMT* transcription in A549 cells following CNPs exposure was observed except for C_60_. However, significance (*P* < 0.05) was only observed in the down-regulation of *DNMT3b* expression following SWCNTs treatment (see ESI Table S6).

## Discussion

When A549 cells are exposed to CNPs, alterations in IR spectra indicate that the dose-response is non-linear except in cells treated with long MWCNTs (Fig. 2). Similarly, spectral datasets from SERS indicate non-linear responses in cells following such exposures (Fig. 5). These non-linear responses are often associated with low-dose effects, which is significant in eco-toxicological assessment as it is more typical of environmental contamination. Biospectroscopy exhibits an ability to detect non-linear responses or low-dose effects in cells^17^,^18^.

However, more pronounced segregation between exposure groups *vs.* control is observed in the SERS-derived scores plot compared to IR (Fig. 5). The IR spectrum is a measurement of absorption after an IR beam is transmitted into the cells. However, the dominant contributor to most SERS processes is the electromagnetic enhancement mechanism; the maximum SERS enhancing region decreases dramatically with distance (*r*^−10^ for spheres), and the largest enhancement is found only a few nanometre from the Au nanoparticle surface^24^. This suggests that most of the signal collected employing SERS is derived from entities in close proximity to the surface of the Au nanoparticle, likely the cell membrane. Therefore, SERS is likely to only generate enhanced localised information of cellular components in an information rich manner. However, it is unknown how reproducible or consistent such enhancement of the Raman signal is. Consequently, ATR-FTIR and Raman spectroscopy techniques are mutually complementary.

In order to determine how CNPs alter cells, loadings plots post-PCA of the spectral dataset were employed to highlight the important wavenumbers related to alterations in exposed cells *vs.* control. Both loadings plots derived from the PC1 (Figs 2 and 5) and PC2 (see ESI) space were used, since loadings in these two PC factors explain most of the variance contributing to segregation among groups in 2-D scores plots (see ESI). In each loadings plot, the top eight distinguishing peaks were highlighted as corresponding to the main biomarkers for the dose-response in CNP-treated cells (all wavenumbers and their tentative assignments are listed in ESI). The loadings plots for both IR and SERS spectra suggest that the four different CNPs shared a similar mode of action in altering cells. Datasets derived from IR spectra highlight significant alterations induced by CNPs in Amide I (~1650 cm^−1^), Amide II (~1500 cm^−1^), lipid (~1750 cm^−1^) and protein (~1400 cm^−1^), while small changes in the region of DNA/RNA (*v*_*as*_PO_2_^−^, ~1225 cm^−1^; *v*_*s*_PO_2_^−^, ~1080 cm^−1^) are observed as well (Fig. 2), suggesting that CNPs may have some genotoxicity. Although signals collected following SERS only generate partial information in the cells, significant alterations are observed in lipid and Amide I regions [*v*(C = C), ~1640 cm^−1^; CH_2_ bending, ~1440 cm^−1^], phenylalanine (~1001 cm^−1^) and DNA (~720 cm^−1^) (Fig. 3). All spectral profiles indicate that alterations induced by CNPs are mostly located in the outer region of cells^21^,^30^. It is believed that generation of ROS and subsequent oxidative stress (OS) is the predominant mechanism of nanotoxicity^31^,^32^. Thus, CNPs could directly or indirectly cause genomic damage inside the cell with no necessity for them reaching the nucleus^33^,^34^.

To compare the effects induced by different CNPs, a parallel experiment was carried out in which A549 cells were exposed to a concentration of 0.1 mg/L. Cells were then collected for biospectroscopic analysis, as well as further global DNA methylation assessment. Following application of PCA-LDA, both 3-D and 2-D scores plots from IR and SERS spectra were derived (Fig. 3). IR spectra indicate that long MWCNTs induce the most pronounced alterations in A549 cells, while C_60_ seem to be less harmful; the C_60_-treated group exhibit a relatively high overlap with the control (Fig. 3A,B). Moreover, the 2-D scores plot derived from IR spectra also shows that short MWCNTs and SWCNTs exert marked effects to a similar extent, but possibly through different underlying mechanisms due to significant segregation between these categories (Fig. 5B). This result is consistent with our previous investigations showing that larger-sized CNPs may induce more pronounced alterations^35^,^36^. In contrast to IR spectra, SERS spectra indicate that short MWCNTs induce the most marked responses in cells, followed by SWCNTs, and then long MWCNTs, with large overlap between the C_60_-exposed group and the control, again suggesting that C_60_ NPs have the least effect (Fig. 3D). Both spectral datasets suggest that C_60_ is the least toxic of the four CNPs^37^. In general, cluster vectors derived from IR spectra show that most alterations are located in the lipid, Amide I and protein regions, while the DNA/RNA region is slightly affected. Both IR and SERS spectra highlighted that the most pronounced biochemical alteration in cells after C_60_ exposure is in the lipid region, which could be a result of its spherical shape and its relatively lipophilic properties. Moreover, SERS detected marked alterations in the DNA/RNA region (intensity ~760 cm^−1^) induced by the three types of CNTs.

Beyond the global biochemical information generated by biospectroscopy, global DNA methylation levels in cells treated with CNPs at 0.1 mg/L were measured using HPLC-MS^38^. These results show that global DNA methylation levels were significantly elevated by C_60_ and short MWCNTs treatment, with marked increases also observed following long MWCNTs or SWCNTs exposure (Fig. 6; see ESI Figure S8). Additionally, utilization of quantitative real-time RT-PCR determined how CNPs impact on DNA intracellular methylation processing. As target genes, *DNMTs* are positive regulators, which can mediate DNA methylation by catalysing the transfer of a methyl group to DNA. DNMT1 mainly contributes to maintaining the pre-existing methylation pattern during replication, while DNMT3a and DNMT3b are mostly involved in *de novo* methylation^39^, which is considered to be implicated in cell growth and differentiation, and also in abnormal methylation in tumorigenesis^40^. Generally, a down-regulating tendency in *DNMTs* transcription in A549 cells after CNPs exposure is observed. Only the transcription level of *DNMT3b* was decreased significantly by SWCNTs, in contrast to the observation of increased global DNA methylation levels.

## Conclusion

Herein, biospectroscopy, both ATR-FTIR spectroscopy and SERS, is presented as a novel approach for nanotoxicity assessment, which also provides biochemical information underlying CNPs-induced cellular alterations. Both IR and SERS spectra show that C_60_, long or short MWCNTs, and SWCNTs exert effects in A549 cells and induce alterations in lipid, protein and even genomic regions. Additionally, it was determined that CNPs at 0.1 mg/L reduce *DNMT* expression, but contribute to global genomic hypermethylation; to our knowledge, this is the first demonstration of a CNP-induced epigenomic effect^29^,^41^. Further study is required to determine real-world effects of CNPs for risk assessment.

## Methods

### Chemicals and carbon nanoparticles

All CNPs were purchased from Sigma. Short MWCNTs were >90% pure being 10–15 nm in diameter and 0.1–10 μm in length. Long MWCNTs were >90% pure, and were 110–170 nm in diameter and 5–9 μm in length. C_60_ had a purity >99.5% and particle size of 1 nm. SWCNTs were described as CarboLex AP-grade (the purity of AP-grade products ranges from 50% to 70% by volume); major impurities are carbon nanospheres and carbon-encapsulated catalyst nanoparticles - the diameter was 1.2–1.5 nm. All CNPs were analysed by Raman spectroscopy (Renishaw PLC, Gloucestershire, UK) with a 785 nm laser, and determined to be of high purity. Additionally, images of CNPs were taken using a scanning electron microscope (SEM) [JSM 5600 (JEOL)] (see ESI Figure S1). Bovine serum albumin (BSA) obtained from Sigma was ≥98%. CNPs were dispersed in 1% BSA solution with a 15-min ultrasonication bathed in ice water and stock solutions were made at concentrations of 100 mg/L. CNT solutions were stable and well-dispersed, while C_60_ appeared to agglomerate.

Gold nanoparticles (Au NPs) for SERS were synthesized using trisodium citrate as the reductants (see ESI Figure S5)^42^,^43^; these were prepared and characterised by Dr Li Cui (at Institute of Urban Environment, Xiamen, China).

### Cell culture and CNPs treatment

Human lung epithelial A549 cells were routinely maintained in RPMI 1640 medium at pH 7.2, supplemented with 10% inactivated foetal bovine serum and 1% penicillin/streptomycin and grown in humidified atmosphere supplied with 5% CO_2_ in air at 37 °C. A549 cells were cultured in 60 mm dishes prior to incorporation into experiments. Following this, cells were disaggregated with trypsin (0.05%)/EDTA (0.02%) solution, and were immediately re-suspended in complete medium and seeded in 30 mm dishes. Then cells were grown for 24-h to attach and followed by a further 24-h treatment or without test agents. The four CNPs were introduced into the treatment at concentrations of 0.1 mg/L, 0.01 mg/L or 0.001 mg/L. Each control and each exposure group was conducted in triplicate.

Post-exposure, A549 cells were washed three times with cold PBS, scraped and centrifuged at 1000 g for 3 min. The resulting cell pellets were fixed with 4% formalin in PBS for 30 min. The fixed cells were washed using distilled water and then added to Low-E glass slides (Kevley Technologies, Chesterland, OH, USA), air-dried and stored in desiccator prior to ATR-FTIR spectroscopy. For SERS, the fixed and washed cells were mixed with Au NPs. After vortexing, an aliquot of 10 μL of the mixture were dropped onto a glass slides for SERS measurement.

### Spectrochemical analysis

All A549 cell samples on Low-E slides were interrogated using a Bruker TENSOR 27 FTIR spectrometer (Bruker Optics Ltd., Coventry, UK) equipped with a Helios ATR attachment containing a diamond internal reflection element (IRE). Instrument parameters were set at 32 scans and 8 cm^−1^ resolution. For each slide, 10 IR spectra were acquired at different points across the sample. Prior to starting a new slide, the ATR crystal was cleaned with deionized water and a background was then taken.

SERS spectra were acquired by using a LabRAM Aramis (HORIBA JobinYvon) confocal micro-Raman system equipped with a 1200 g/mm grating, He-Ne 632.8 nm laser (laser power ≤70 mW prior to lens). The system calibration was carried out using a silicon calibration source for wavenumber shift. A 50× objective (Olympus) with a numerical aperture of 0.55 was used to focus the laser beam and collect Raman signal with a working distance of about 8 mm. In order to reduce any laser damage to cells, DuoScan in the micromapping mode with a scanning area of 30 μm × 30 μm was applied with an acquisition time of 5 sec. For each sample, 5 spectra were acquired.

### Computational analysis of spectral data

Spectral data processing, acquired from both ATR-FTIR and Raman spectroscopy, were performed using IRootLab toolbox ( http://irootlab.googlecode.com) running on MATLAB r2010a (The MathWorks, Inc., US)^44^. IR spectra were pre-processed as followings: cut to 1800–900 cm^−1^ (the biochemical fingerprint range), 2^nd^ differentiation and vector normalisation. SERS spectra were initially processed in LabSpec 6 (software provided with the Raman system) for cosmic ray removal and background correction (see ESI Figure S6). The spectral data were then inputted into MATLAB and pre-processed following wavelet de-noising, cut to 400–1800 cm^−1^, 2^nd^ differentiation, and vector normalisation^45^. The differentiation is employed both as a means of baseline correction and to resolve overlapped bands^45^. The wavelet de-noising utilizes non-linear filtering implemented through multi-scale decomposition and thresholding for de-noising, especially for the spectra containing sharp peaks. Computational analysis using multivariate techniques including PCA and LDA can efficiently analyse such large spectral datasets^46^,^47^. The main difference between PCA and LDA is that the former is an unsupervised method, whilst the latter is supervised. PCA looks for projections to maximize variance and LDA looks for projections that maximize the ratio of between-category to within-category scatter^48^. Following pre-processing, PCA was applied to the dataset. PCA reduces the dimensions of the data. Undoubtedly PCA is capable of identifying important information in spectral data, but has less discriminatory power. Often, in order to interpret complex biochemical information with labelled classes, further analysis using supervised procedures like LDA is required. The output data derived from PCA or PCA-LDA (the first ten PC factors of PCA are used for LDA since >99% of variance is captured) can then be visualized as 1-D, 2-D or 3-D scatterplots (“scores plots”). In scores plots, nearness between two categories implies similarity, while distance indicates dissimilarity. To reveal the biochemical alterations associated with each category in the dataset, both loadings plots and cluster vectors^16^ were developed. To simplify the identification of the main biochemical alterations distinguishing each category, peak detector was used to indicate the first few highest peaks in the loadings plots or cluster vectors plots.

### Global DNA methylation determined by HPLC-MS

A549 cells exposed to four CNPs at 0.1 mg/L were collected and stored in PBS at −20 °C prior to quantitative real-time RT-PCR or DNA methylation analysis. DNA was extracted from A549 cells using the DNeasy Cell Kit (Qiagen, Germany) following the manufacturer’s instructions. RNase A was added to columns in the kit to remove RNA residue. DNA hydrolysis was conducted using a mixture degradase kit (DNA Degradase Plus, Zymo Research, USA) following the manufacturer’s protocol. To confirm complete hydrolysis of DNA, agarose gel electrophoresis was employed to test the result of the hydrolysis (see ESI Figure S7). The DNA hydrolysis mixtures were then stored at −20 °C for mass spectrometric analysis.

To perform chromatographic separation, a Kinetex C_18_ column (100 mm × 4.6 mm, 2.6 μm; Phenomenex, USA) was employed in a HPLC system (LC-20AD, Shimadzu, Japan). The injection volume was 20 μL. The mobile phase consisted of water (A) and methanol (B). A gradient elution protocol was as follows: 0–0.01 min, 3% B; 0.01–5.00 min, 5% B; 3.00–12.00 min, 50% B; 12.00–15.00 min, 100% B; 15.00–25.00 min, 3% B at a flow rate of 0.5 mL.min^−1^. For the mass spectrometric analysis, an electrospray ionization tandem mass spectrometry (LCMS-8030, Shimadzu, Japan) system was used, operating in positive ionization mode and conditioned at a capillary temperature of 400 °C and medium N_2_ curtain gas. Optimized multiple reaction monitoring (MRM) conditions were set to evaluate dC from m/z 228.1 to 111.9 and 5 mdC from m/z 242.1 to 126.0. Data acquisition and processing were performed *via* Analyst software. The global DNA methylation ratio (MR) was determined by MR = [5 − mdC]/([5 − mdC] + [dC]).

### Quantitative real-time RT-PCR

Total RNAs were extracted from cells using the RNeasy^@^ Mini Kit (Qiagen, Venlo, Netherlands). Subsequently, reverse transcription of cDNA synthesis was performed with 1 μg total RNA using PrimeScript^@^ RT reagent kit with gDNA Eraser cDNA synthesis kits (Takara, Otsu, Shiga, Japan). Real-time PCR was carried out in a 20 μL reaction mixture in triplicate, using SYBR Green Master Mix reagents (Roche, Basel, Switzerland) on a Roche LightCycle^@^ 480 ll real-time PCR system following the manufacturer’s protocol (95 °C for 10 min followed by 40 cycles at 95 °C for 15 sec, and 60 °C for 30 sec). The primer sequences specific for *DNMT1*, *DNMT3a*, *DNMT3b* and *GAPDH* were designed using the primer5 software (see ESI Table S7). Gene expression levels were normalised to *GAPDH* expression. The relative levels of each target mRNA transcripts to the control *GAPDH* were analysed by 2^−ΔΔCt^ method and expressed as fold change.

### Statistical analysis

The data are expressed as the mean ± SD. Significant differences among multiple groups were determined using a one-way analysis of variance (ANOVA) followed by Dunnett’s *post hoc* tests. Probabilities of *P* < 0.05 were considered as statistically significant. All these tests were conducted in GraphPad Prism 4 (GraphPad Software, USA).

## Additional Information

**How to cite this article**: Li, J. *et al.* Low-dose carbon-based nanoparticle-induced effects in A549 lung cells determined by biospectroscopy are associated with increases in genomic methylation. *Sci. Rep.* **6**, 20207; doi: 10.1038/srep20207 (2016).

## Supplementary Material

Supplementary Information

## Figures and Tables

**Figure 1:**
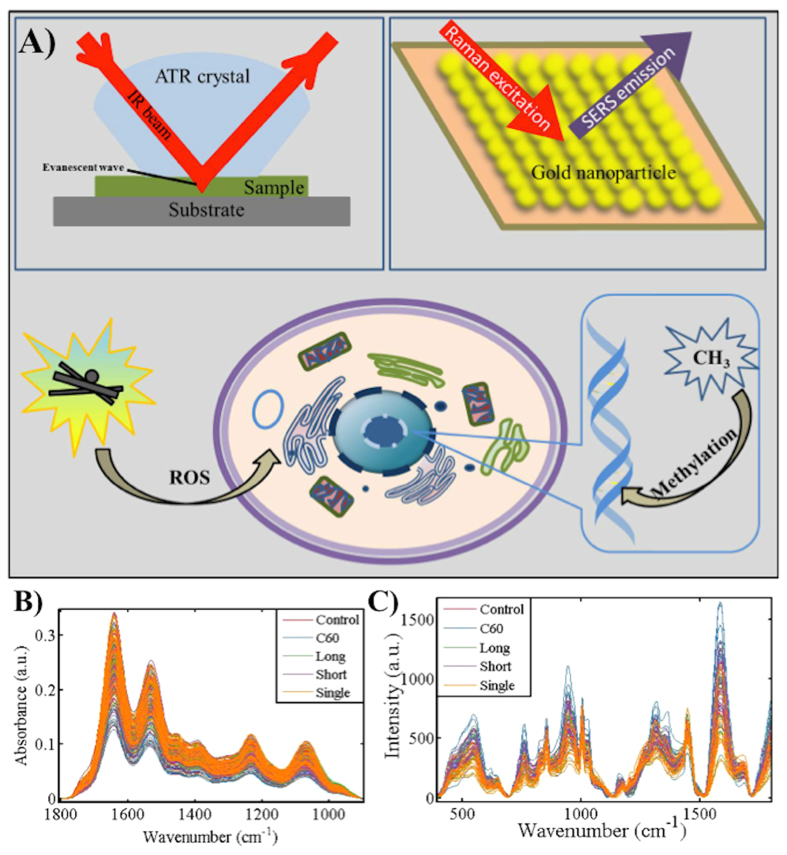
Experimental overview of application of biospectroscopy techniques towards nanotoxicology testing. (**A**) General schematic for brief introduction of nanotoxicity and the basic principle of attenuated total reflection Fourier-transform infrared (ATR-FTIR) spectroscopy and surface-enhanced Raman spectroscopy (SERS) for this experimental approach (**B**) Raw spectra in the “biochemical fingerprint region” from A549 cells following ATR-FTIR spectroscopy; and (**C**) Raw spectra of A549 cells following SERs.
