# Supplementary material for: Low-dose carbon-based nanoparticle-induced effects in A549 lung cells determined by biospectroscopy are associated with increases in genomic methylation
Source: Sci Rep. 2016 Feb 2;6:20207. doi: 10.1038/srep20207 (PMC4735790; doi:10.1038/srep20207)
Supplement: Supplementary Information [file srep20207-s1.pdf]

Electronic Supporting Information

Low-dose effects induced by carbon-based nanoparticles in A549 lung cells determined by biospectroscopy are associated with increases in genomic methylation

*Junyi Li[a], Meiping Tian[b], Li Cui[b], John Dwyer,[c] Nigel J. Fullwood,[c] Heqing Shen[b] and Francis L. Martin[a]**

[a]Centre for Biophotonics, LEC, Lancaster University, Lancaster LA1 4YQ, UK; [b]Key Lab of Urban Environment and Health, Institute of Urban Environment, Chinese Academy of Sciences, Xiamen 361021, China; and, [c]Division of Biomedical and Life Sciences, Faculty of Health and Medicine, Lancaster University, Lancaster LA1 4YQ, UK

***Corresponding author** email: f.martin@lancaster.ac.uk; Tel: +44 (0)1524 510206

Figure S1 Scanning electron microscopy (SEM) images of **(a)** C_{60} fullerene (Scale bar = 10 μm); **(b)** long MWCNTs (Scale bar = 2 μm); **(c)** short MWCNTs (Scale bar = 0.5 μm), and **(d)** single-walled CNTs (Scale bar = 0.2 μm); Raman spectral insets representative of typical characterization.

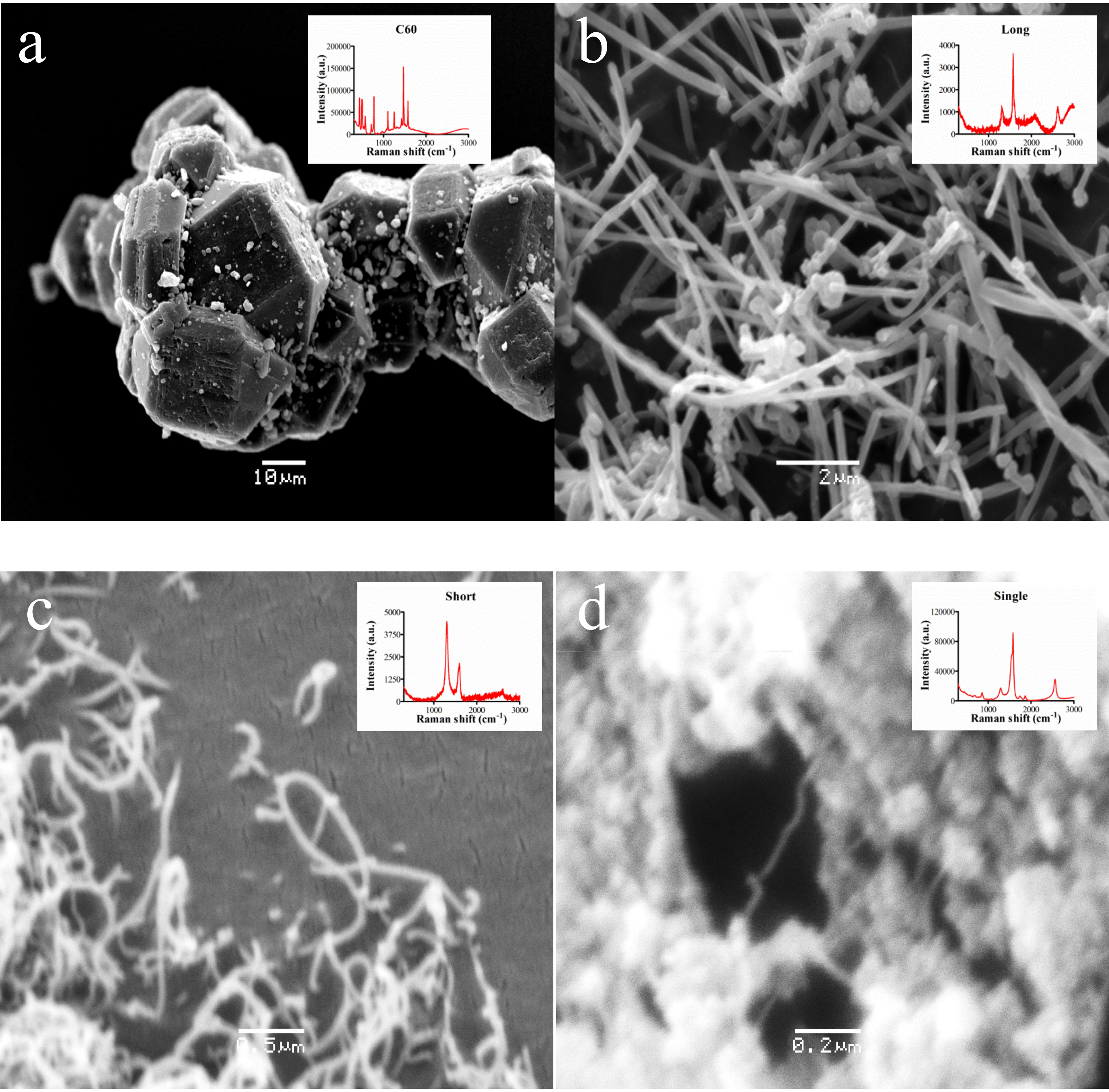

Figure S2 Two-D **PCA** scores plots (*90% Confidence ellipsoids*) and loadings plots in PC2 derived from A549 cells exposed to carbon nanoparticles (CNPs) interrogated by **ATR-FTIR spectroscopy**.
a) C_{60}; **b**) long MWCNTs; **c**) short MWCNTs; and, **d**) single-walled CNTs.

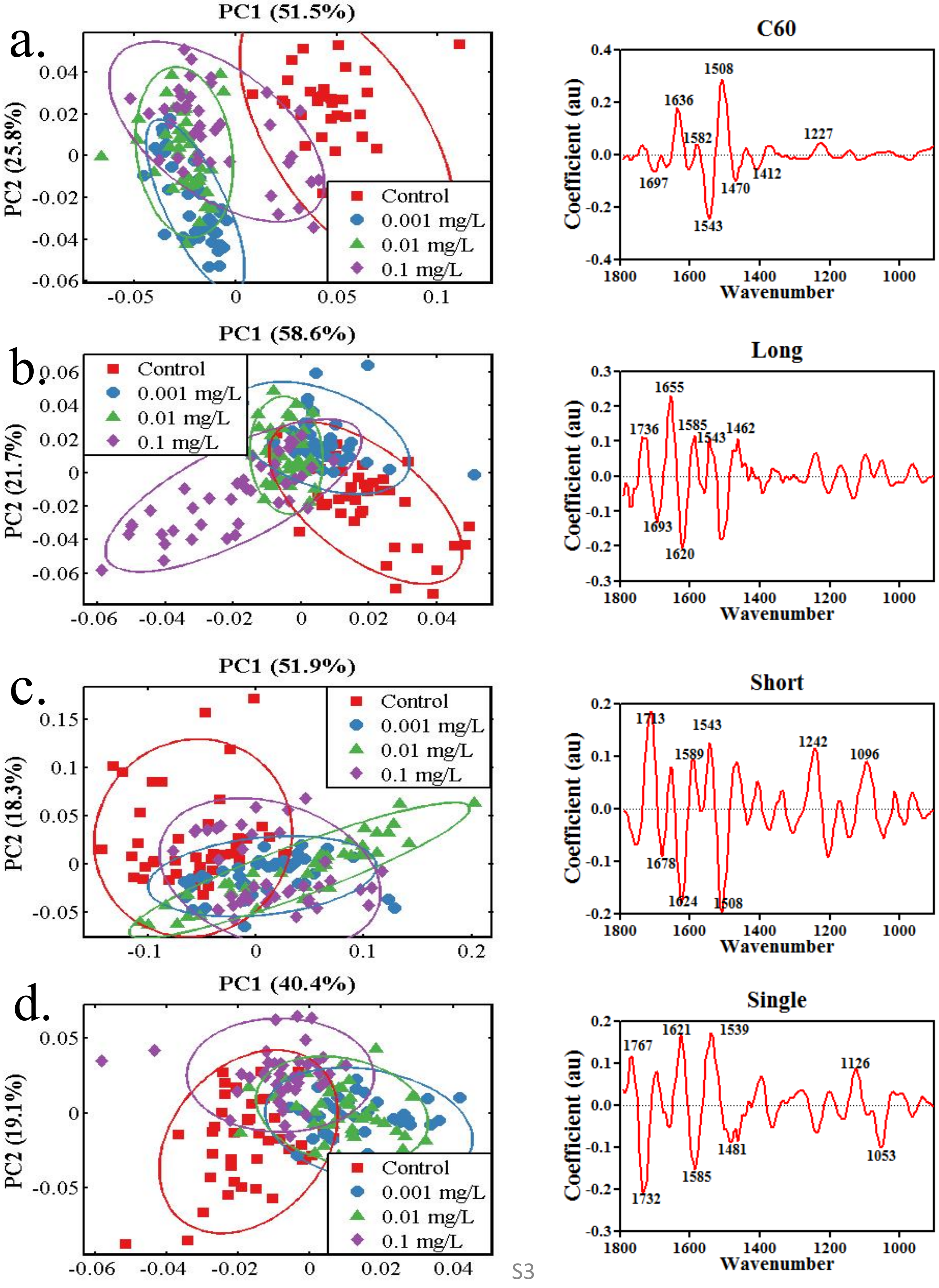

Figure S3 Two-D **PCA** scores plots (*90% Confidence ellipsoids*) and loadings plots in PC2 derived from A549 cells exposed to CNPs interrogated by **SERS**. **a**) C_{60}; **b**) long MWCNTs; **c**) short MWCNTs; and, **d**) single-walled CNTs.

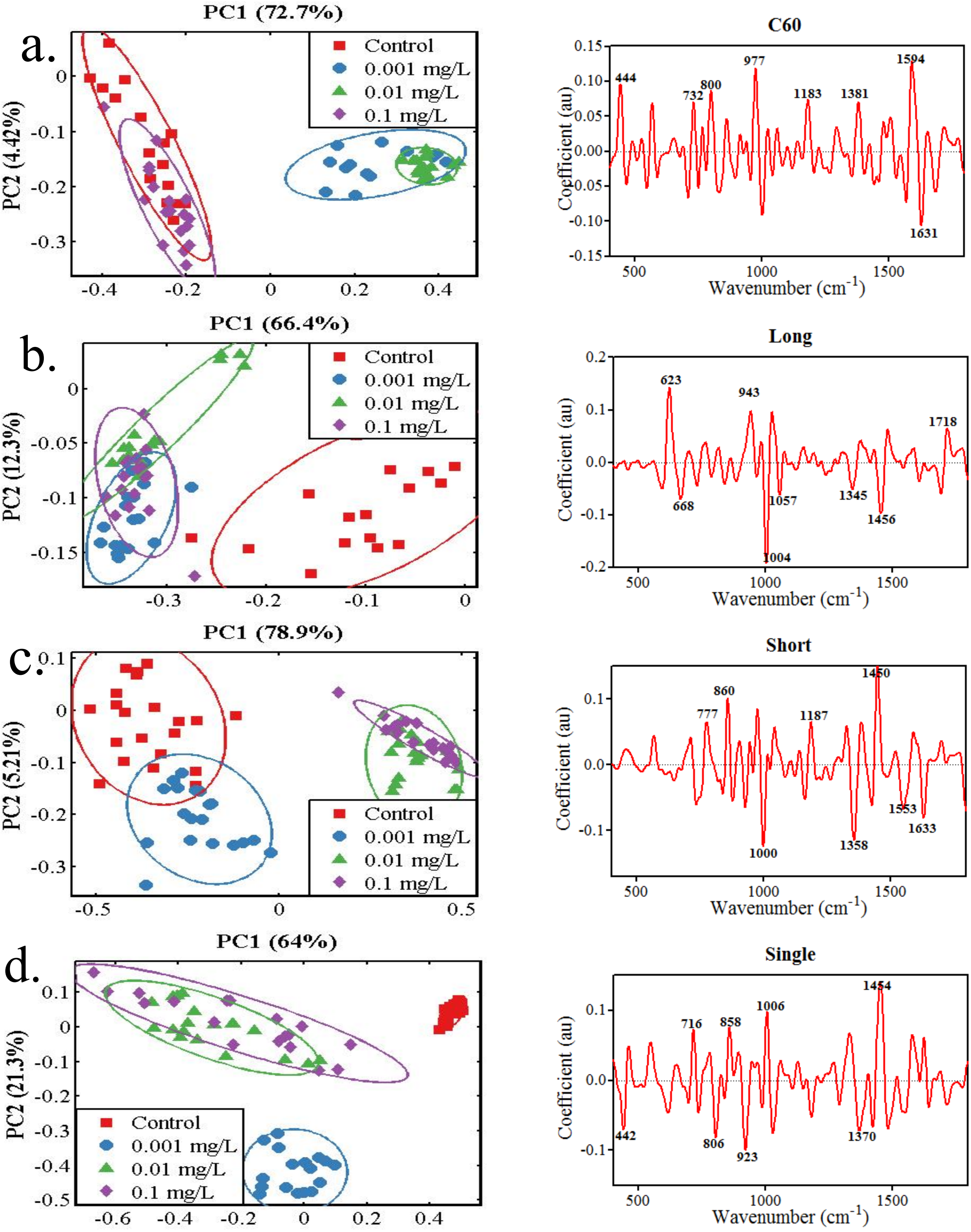

Figure S4. Cluster vectors indicating the wavenumber basis for segregation of A549 cells exposed to different carbon nanoparticles (CNPs) at 0.1 mg/L. Cells exposed to CNPs at 0.1 mg/L were compared to vehicle control. The height of the cluster vector peak is proportional to the extent of biochemical alteration compared to the vehicle control.
Cells were interrogated by: **a**) ATR-FTIR spectroscopy; or, **b**) SERS.
C60, C_{60} fullerene; Long, long MWCNTs; Short, short MWCNTs; Single, SWCNTs

a.

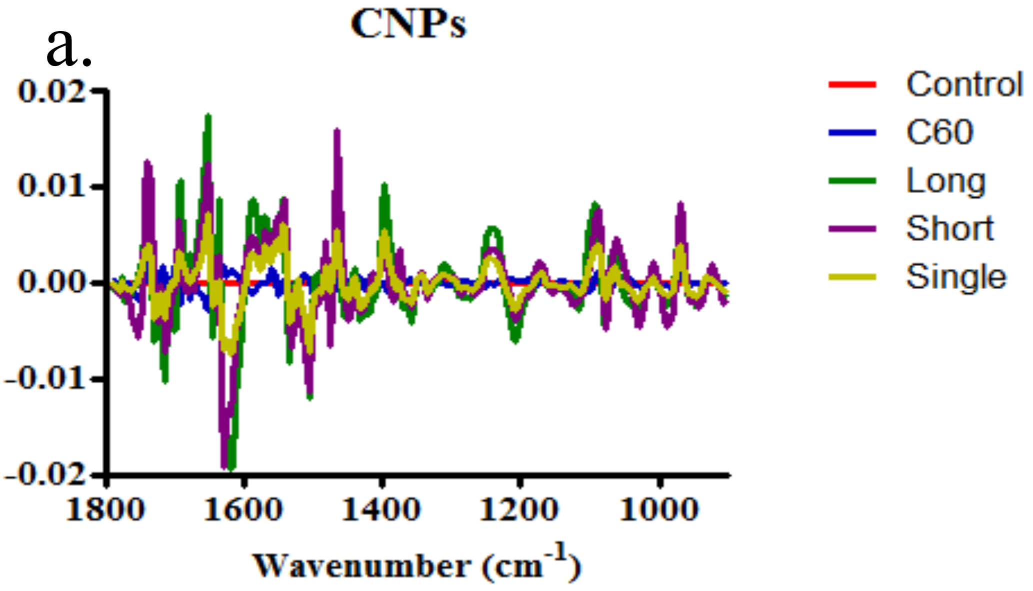

b.

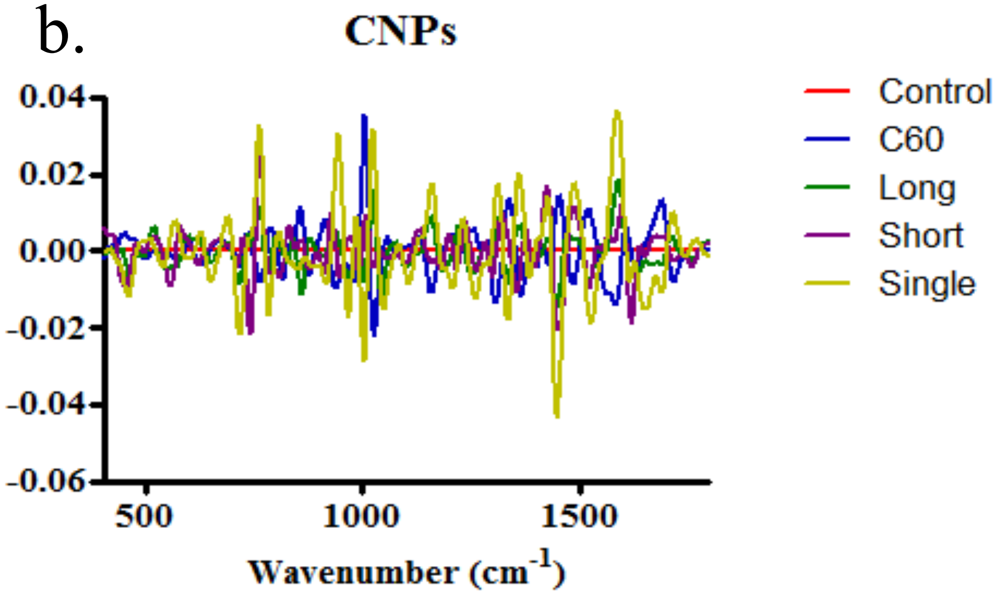

Figure S5

Synthesis of Au nanoparticles (NPs). Au NPs were prepared according to Frens' method.

Briefly,100 mL of 0.01% (wt/vol) $HAuCl_4$ aqueous solution was heated to boiling under vigorous stirring, followed by the immediate addition of 0.6 mL of 1% (wt/vol) trisodium citrate solution. The solution was kept boiling for about 1 h. Then the Au NPs solution was allowed to cool down for subsequent application.
Prior to being applied to the cell samples, Au NPs solution were washed with de-ionised water twice and concentrated using centrifugation.

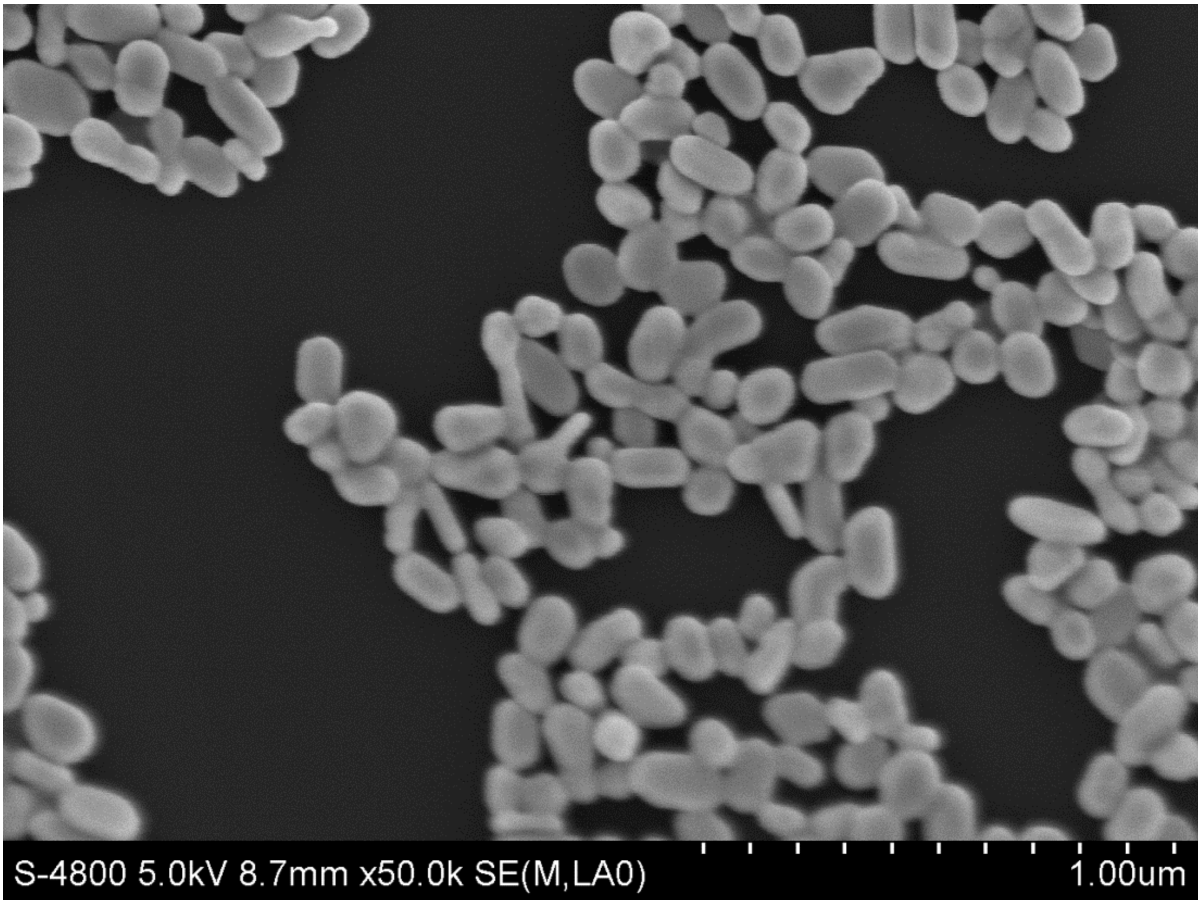

Figure S6

a) Raw spectra of SERS**;** and, **b)** Spectra of SERS after cosmic ray removal and background correction in LabSpec 6.

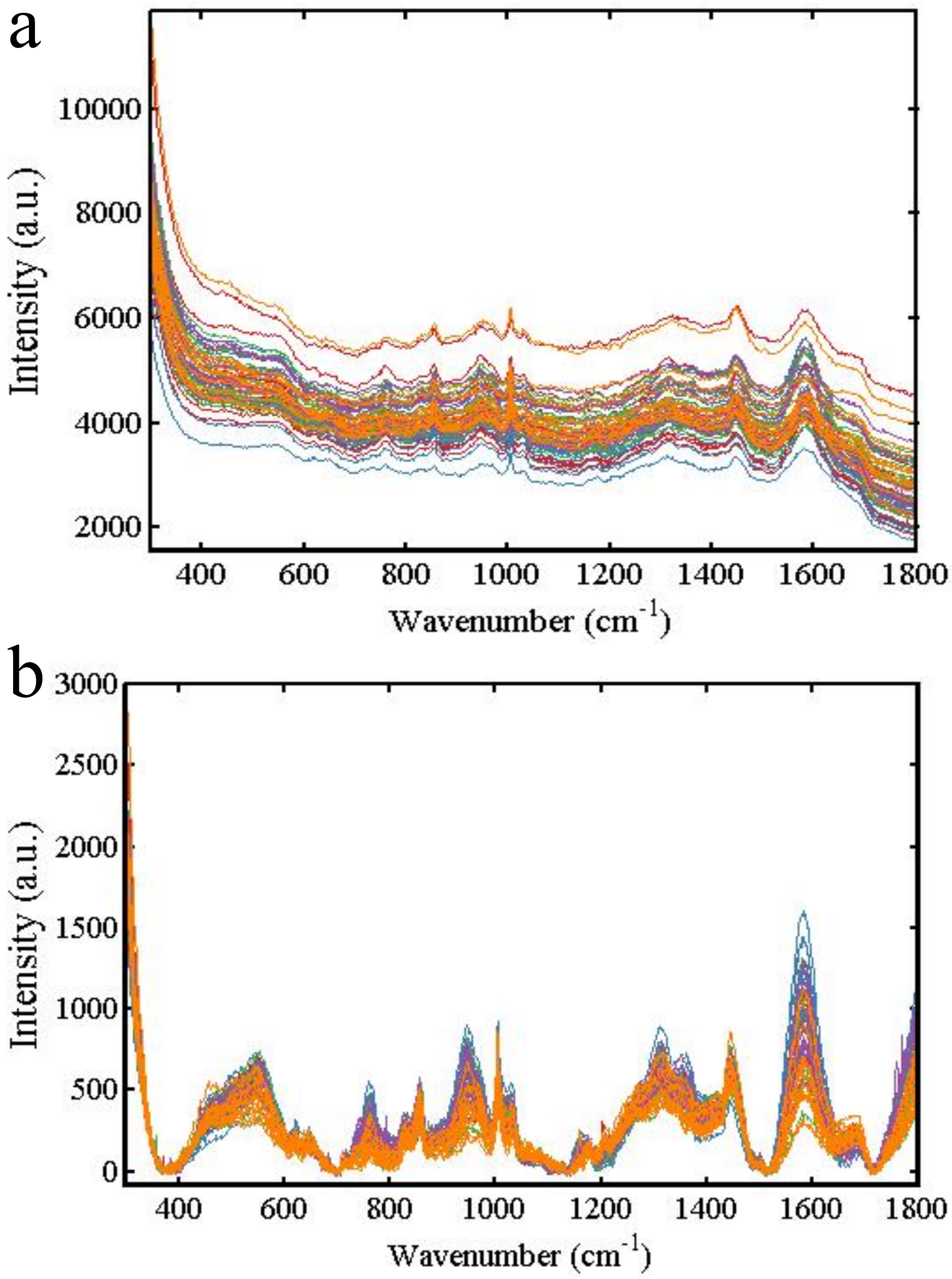

Figure S7

Contrast of the results of DNA extraction and DNA digestion by enzymes: (1) DNA extracted from cells; (2) DNA digested by enzymes; and, (3) Marker： 100 bp DNA ladder.

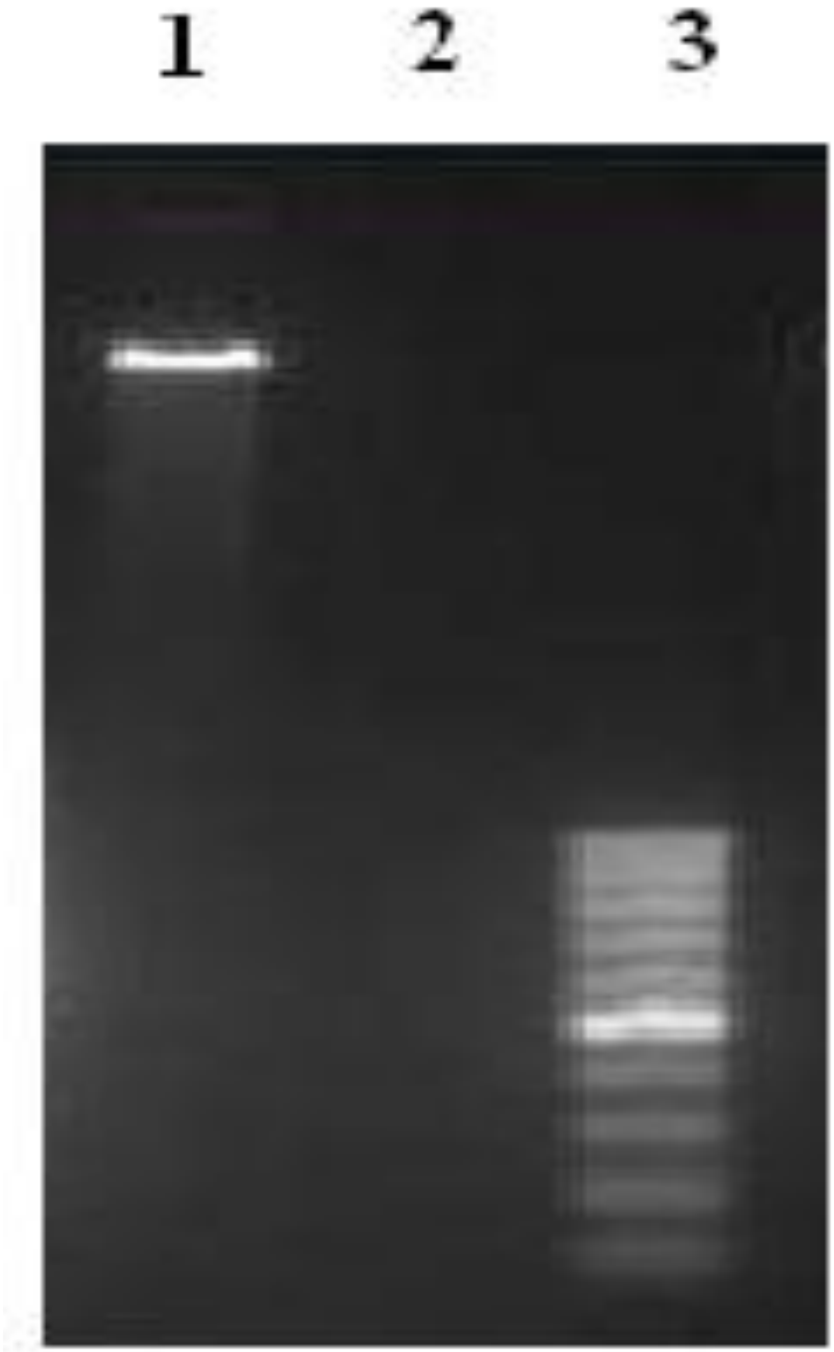

Table S1

Primary wavenumbers in loadings plots derived from PCA of ATR-FTIR spectral dataset

	PC1		PC2	
	Wavenumber (cm^{-1})	Tentative assignments	Wavenumber (cm^{-1})	Tentative assignments
**C_{60}**	1624	Amide I	1508	Amide II
	1659	Amide I	1543	Amide II
	1589	Amide I	1636	Amide I
	1547	Amide II	1470	Proteins, $v_{as}CH_3$
	1493	Proteins, $v_{as}CH_3$	1697	Lipid, v(C=O)
	1709	Lipid, v(C=O)	1412	$v(COO^-)$
	1396	$v(COO^-)$	1227	$v_{as}PO_2^-$
	1103	$v_sPO_2^-$	1582	Amide II
**Long MWCNTs**	1539	Amide II	1655	Amide I
	1501	Amide II	1620	Amide I
	1624	Amide I	1508	Amide II
	1585	Amide I	1693	Amide I
	1663	Amide I	1585	Amide I
	1466	Proteins, $v_{as}CH_3$	1736	Lipid, v(C=O)
	1732	Lipid, v(C=O)	1543	Amide II
	1065	$v_sPO_2^-$	1462	Proteins, $v_{as}CH_3$
**Short MWCNTs**	1620	Amide I	1508	Amide II
	1547	Amide II	1713	Lipid, v(C=O)
	1501	Amide II	1624	Amide I
	1655	Amide I	1543	Amide II
	1585	Amide I	1242	$v_{as}PO_2^-$
	1462	Proteins, $v_{as}CH_3$	1204	$v_{as}PO_2^-$
	1717	Lipid, v(C=O)	1589	Amide I
	1400	$v(COO^-)$	1678	Amide I
**Single-walled CNTs**	1508	Amide II	1732	Lipid, v(C=O)
	1466	Proteins, $v_{as}CH_3$	1539	Amide II
	1678	Amide I	1624	Amide I
	1732	Lipid, v(C=O)	1585	Amide I
	1061	$v_sPO_2^-$	1767	Lipid, v(C=O)
	1582	Amide II	1053	Glycogen
	1018	Glycogen	1126	v(CO)
	1114	v(CO)	1481	Proteins, $v_{as}CH_3$

Table S2

Primary wavenumbers in loadings plots derived from PCA of SERS spectral dataset

	PC1		PC2	
	Wavenumber (cm^{-1})	Tentative assignments	Wavenumber (cm^{-1})	Tentative assignments
**C60**	1454	Lipid, CH_2	1594	Lipid, C=C
	1006	Phenylalanine	977	Protein
	930	Protein	1631	Lipid, C=C
	1364	Tryptophan, protein	444	Glycogen
	1621	Lipid, C=C	800	DNA, nucleic acid
	1423	Lipid, CH2	1183	Protein
	858	Collagen	1382	Tryptophan, protein
	1487	Lipid, CH_2	732	DNA, nucleic acid
**Long MWCNTs**	1055	Protein	1004	Phenylalanine
	518	*v*(S-S)	623	*v*(C-S)
	1586	*v*(C=C)	944	Protein
	463	Glycogen	1456	Lipid, CH_2
	761	DNA, nucleic acid	668	Protein, *v*(C-S)
	1522	-C=C-, Amide II	1718	Lipid, *v*(C=O)
	625	Protein, *v*(C-S)	1057	Collagen
	1335	Lipid/protein, CH_3/CH_2	1345	Lipid/protein, CH_3/CH_2
**Short MWCNTs**	1006	Phenylalanine	1450	Lipid, CH_2
	1456	Lipid, CH_2	1000	Phenylalanine
	1341	Lipid/protein, CH_3/CH_2	1358	Lipid/protein, CH_3/CH_2
	1372	Tryptophan, protein	860	Collagen
	765	DNA, nucleic acid	1633	Lipid, C=C
	946	Protein	777	DNA, nucleic acid
	1718	Lipid, *v*(C=O)	1553	-C=C-, Amide II
	1623	Lipid, C=C	1187	Protein
**Single-walled CNTs**	1623	Lipid, C=C	1454	Lipid, CH_2
	442	Glycogen	923	Protein
	1179	Protein	1006	Phenylalanine
	801	DNA, nucleic acid	806	DNA, nucleic acid
	938	Protein	858	Collagen
	1030	Phenylalanine	716	DNA, nucleic acid
	1417	Lipid, CH_2	1370	Tryptophan, protein
	728	DNA, nucleic acid	442	Glycogen

Table S3

Cluster vector peaks plot derived from PCA-LDA of spectral dataset

	ATR-FTIR		SERS	
	Wavenumber (cm^{-1})	Tentative assignments	Wavenumber (cm^{-1})	Tentative assignments
**C60**	1732	Lipid, v(C=O)	1590	v(C=C)
	1663	Amide I	1004	Phenylalanine
	1462	Proteins, $v_{as}CH_3$	765	DNA, nucleic acid
	1628	Amide I	1308	Lipid/protein, CH_3/CH_2
	1092	$v_sPO_2^-$	1524	-C=C-, Amide II
	1547	Amide II	1366	Tryptophan, protein
	972	Protein phosphorylation	946	Protein
**Long MWCNTs**	1620	Amide I	1596	C=N/C=C, protein
	1659	Amide I	761	DNA, nucleic acid
	1582	Amide II	1047	Glycogen
	1242	$v_{as}PO_2^-$	1313	Lipid/protein, CH_3/CH_2
	1204	$v_{as}PO_2^-$	1525	-C=C-, Amide II
	1516	Amide II	716	DNA, nucleic acid
	1099	$v_sPO_2^-$	1160	Protein
**Short MWCNTs**	1620	Amide I	759	DNA, nucleic acid
	1736	Lipid, v(C=O)	1621	Lipid, C=C
	1462	Proteins, $v_{as}CH_3$	1428	Lipid, CH_2
	1655	Amide I	1008	Phenylalanine
	1508	Amide II	928	Protein
	1558	Amide II	1526	-C=C-, Amide II
	1238	$v_{as}PO_2^-$	726	DNA, nucleic acid
**Single-walled CNTs**	1620	Amide I	761	DNA, nucleic acid
	1508	Amide II	1594	C=N/C=C, protein
	1543	Amide II	944	Protein
	1655	Amide I	1020	Phenylalanine
	1242	$v_{as}PO_2^-$	1448	Lipid, CH_2
	1096	$v_sPO_2^-$	1530	-C=C-, Amide II
	1462	Proteins, $v_{as}CH_3$	716	DNA, nucleic acid

Table S4.

Scores plots in PC1 space following PCA of spectra derived from A549 cells exposed to CNPs					
One-way ANOVA with Dunnett's Multiple Comparison Test		C_{60}	Long MWCNTs	Short MWCNTs	Single-walled CNTs
**ATR-FTIR spectroscopy**	Control *vs.* 0.001 mg/L	$P < 0.001$	$P < 0.001$	$P < 0.001$	$P < 0.001$
	Control *vs.* 0.01 mg/L	$P < 0.001$	$P < 0.001$	$P < 0.001$	$P < 0.001$
	Control *vs.* 0.1 mg/L	$P < 0.001$	$P < 0.001$	$P < 0.001$	$P < 0.001$
**SERS**	Control *vs.* 0.001 mg/L	$P < 0.001$	$P < 0.001$	$P < 0.001$	$P < 0.001$
	Control *vs.* 0.01 mg/L	$P < 0.001$	$P < 0.001$	$P < 0.001$	$P < 0.001$
	Control *vs.* 0.1 mg/L	$P < 0.05$	$P < 0.001$	$P < 0.001$	$P < 0.001$

Table S5.

Global DNA methylation levels of A549 cells exposed to CNPs (0.1 mg/L) assessed by HPLC-MS				
One-way ANOVA with Dunnett's Multiple Comparison Test	C_{60}	Long MWCNTs	Short MWCNTs	Single-walled CNTs
Control *vs.* CNP	$P < 0.05$	$P > 0.05$	$P < 0.05$	$P > 0.05$

Table S6.

**Relative mRNA level of A549 cells exposed to CNPs (0.1 mg/L) assessed by quantitative real-time RT PCR**					
One-way ANOVA with Dunnett's Multiple Comparison Test		C_{60}	Long MWCNTs	Short MWCNTs	Single-walled CNTs
DNMT1	Control *vs*. CNP	*P* >0.05	*P* >0.05	*P* >0.05	*P* >0.05
DNMT3a	Control *vs*. CNP	*P* >0.05	*P* >0.05	*P* >0.05	*P* >0.05
DNMT3b	Control *vs*. CNP	*P* >0.05	*P* >0.05	*P* >0.05	*P* <0.05

Table S7.

Sequence of primers used for quantitative real-time RT-PCR		
Gene	Primer sequence (5´-3´)	Product size (bp)
DNMT1	F: TACCTGGACGACCCTGACCTC	103
	R: CGTTGGCATCAAAGATGGACA	
DNMT3a	F: TATTGATGAGCGCACAAGAGAGC	111
	R: GGGTGTTCCAGGGTAACATTGAG	
DNMT3b	F: GGCAAGTTCTCCGAGGTCTCTG	113
	R:TGGTACATGGCTTTTCGATAGGA	
GAPDH	F:GGAGAAGGCTGGGGCTCAT	230
	R: TGATGGCATGGACTGTGGTC	
